# Sarcopenia Index Is Correlated with Osteoporosis in Patients with Chronic Kidney Disease

**DOI:** 10.3390/diagnostics15010096

**Published:** 2025-01-03

**Authors:** Segi Kim, Simho Jeong, Kyeongmi Kim, Junhee Sung, Do Kyung Kim, Soonchul Lee

**Affiliations:** 1 Department of Orthopaedic Surgery, CHA Bundang Medical Center, School of Medicine, CHA University, Seongnam 13496, Republic of Korea; a196021@chamc.co.kr (S.K.); a226020@chamc.co.kr (S.J.); myjun08@naver.com (J.S.); 2 Department of Laboratory Medicine, CHA Ilsan Medical Center, School of Medicine, CHA University, 100, Ilsan-ro, Ilsandong-gu, Goyang 10414, Republic of Korea; kmi0905@chamc.co.kr; 3 CHA Graduate School of Medicine, 120 Hyeryong-ro, Pocheon 13496, Republic of Korea; 4 Personal Beauty GU Clinic, Seoul 06614, Republic of Korea; 5 SL Bio, Inc., 43, Beolmal-ro 30beon-gil, Bundang-gu, Seongnam 13503, Republic of Korea

**Keywords:** sarcopenia index, osteoporosis, chronic kidney disease

## Abstract

**Objectives:** This study aimed to investigate the relationship between the sarcopenia index (SI), which is derived from serum creatinine and cystatin C levels, and osteoporosis in chronic kidney disease (CKD). **Methods:** This study initially included patients who underwent dual-energy X-ray absorptiometry (DXA) and serum creatinine and cystatin C testing between 2005 and 2022. Subsequently, patients diagnosed with CKD were selected for the final analysis, totaling 102 patients. Both traditional and new SI were calculated, with each participant categorized into one of two groups (non-osteoporosis and osteoporosis) according to bone mineral density. To enhance statistical validity, the patients were further divided into low- and high-index groups based on the median value of both indices for comparative analysis. The association between SI and the risk of osteoporosis was estimated using multivariable logistic regression analysis. **Results:** Participants with lower SI values had lower bone mineral density and a higher diabetes mellitus prevalence. The non-osteoporotic group exhibited significantly higher mean values for both traditional and new SI. Multivariable logistic regression analysis identified three statistically significant variables: both indices, sex, and diabetes mellitus. Both traditional and new SI yielded individual odds ratios of 0.906 with estimated areas under the curve of 0.847 for traditional SI and 0.833 for new SI. **Conclusions:** This study confirmed that both traditional and new SI are associated with osteoporosis in patients with CKD. Therefore, clinicians can raise the suspicion of osteoporosis based on traditional and new SI in patients with CKD, even when DXA results are unavailable.

## 1. Introduction

Osteoporosis is a systemic skeletal disorder characterized by diminished bone mass and microstructural deterioration of the bone tissue, leading to an increased susceptibility to fractures. Sarcopenia is an age-related reduction in skeletal muscle mass [1]. Chronic kidney disease (CKD) is defined as kidney damage or decreased kidney function (measured by glomerular filtration rate (GFR) which remains below 60 mL/min/1.73 m^2^) for 3 months or more, regardless of the cause. CKD affects the kidney’s ability to filter waste, maintain mineral balance, produce red blood cells, and regulate blood pressure [2]. CKD and osteoporosis are closely linked because of their roles in mineral metabolism. As kidney function declines, the body struggles to maintain appropriate calcium and phosphorus levels, which are crucial for bone health [3,4]. Additionally, CKD often leads to increased parathyroid hormone (PTH) production, which can cause bone loss [3,4]. The kidneys also play a role in activating vitamin D, which is essential for calcium absorption. CKD can lead to vitamin D deficiency, which contributes to bone weakening [3,4]. Together, these and other factors contribute to CKD mineral and bone disorder, a condition that substantially elevates the risk of osteoporosis and fractures among patients with CKD [2,3].

Patients with CKD frequently undergo blood tests, including cystatin C and creatinine measurements, as both are important markers used to measure GFR. Although creatinine is a traditional marker, cystatin C is less affected by muscle mass and dietary factors, potentially providing a more accurate GFR estimate in certain populations [5]. By monitoring these laboratory values, healthcare providers can better assess kidney function, adjust treatment plans, and potentially slow the progression of CKD and its associated complications.

In 2017, Kashani et al. introduced a sarcopenia index (SI), referred to as the traditional SI in this paper, to estimate skeletal muscle mass, utilizing serum creatinine and cystatin C [6]. This index has demonstrated a positive correlation with muscle mass and strength across diverse populations, including patients in intensive care units [7], individuals with type 2 diabetes mellitus (DM) or CKD [8,9], and the general population [10]. Additionally, investigations utilizing the SI have identified its association with the prognosis of various diseases. Notably, it has been linked to recurrent stroke and mortality [11], prognostic implications in cardiovascular disease [12], outcomes in patients experiencing acute exacerbations of chronic obstructive pulmonary disease [13,14], and survival rates in hospitalized older patients [14]. Recently, Lien proposed a new SI based on serum creatinine and cystatin C-based GFR [15].

Both sarcopenia indices (traditional and new SI) are derived from serum creatinine and cystatin C values, which are commonly measured in patients with CKD and recognized for reflecting sarcopenia and other diseases. However, their potential association with osteoporosis remains underexplored. Therefore, this study aimed to investigate whether both indices (traditional and new SI) are associated with osteoporosis.

## 2. Methods

### 2.1. Patients

**Figure 1:**
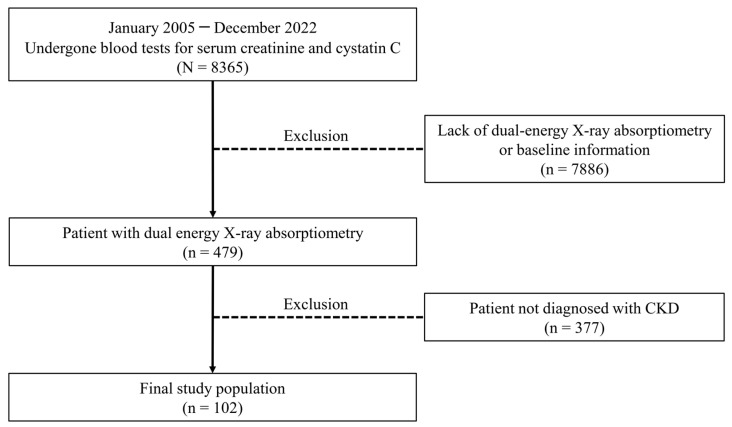
Study population.

The study protocol was reviewed and approved by the Institutional Review Board of the CHA Bundang Medical Center, Korea. (No. 2024-02-015). Informed consent was waived by this committee because of the retrospective nature of our study. This retrospective study was conducted at a single institution, involving patients who sought medical care at CHA Bundang Medical Center between 1 January 2005 and 31 December 2022, and had undergone blood tests for serum creatinine and cystatin C. From this pool, we identified individuals who had also undergone dual-energy X-ray absorptiometry (DEXA), resulting in a final cohort of 479 patients. Of these, 102 patients (Figure 1), for whom both the SI (referred to herein as traditional SI) and new SI were calculated using creatinine, cystatin C, and cystatin C-based GFR, were included in the study.

### 2.2. SI and Other Variables

The calculation method adhered to the protocols proposed in previous studies [6,15].

- Traditional SI = Serum Creatinine [mg/dL]/Serum Cystatin C [mg/L] × 100
- New SI = Serum Creatinine [mg/dL] × Cystatin C-based estimated GFR (eGFR_CysC) [mL/min/1.73 m^2^]

Patients were divided into two groups based on the median values of the traditional SI (median: 94.75) and new SI (median: 78.75) to statistically validate the relationship between SI and osteoporosis. These groups were designated the low SI and high SI groups, respectively, for statistical analysis. Patient demographics, including age, sex, and body mass index (BMI), were recorded. Additionally, comorbidities such as hypertension, DM, hyperlipidemia, cerebrovascular accident, liver cirrhosis, angina, asthma, cancer, and hemodialysis were examined in individuals with chronic renal failure. Based on the DEXA scan, bone mineral density (BMD) T-score was assessed in both the lumbar spine (L1–L4) and proximal femoral region (femur neck and total hip) using DEXA with the Lunar Prodigy Advance system (GE Lunar, Madison, WI, USA). Detailed values of serum creatinine (mg/dL) and serum cystatin C (mg/L) were obtained from venous blood and measured using a Cobas 8000 c702 analyzer (Roche Diagnostics System, Basel, Switzerland).

Participants were categorized into two groups: the non-osteoporosis and osteopenia group (T-score above −2.5) and the osteoporosis group (T-score of −2.5 or below), comprising 54 and 48 patients, respectively.

### 2.3. Statistical Analysis

Various statistical tests were used to compare the two groups, including Pearson’s chi-square test, Mann–Whitney U test, and *t*-test. A logistic regression analysis was conducted to identify variables with significant influence, and a receiver operating characteristic (ROC) curve was generated by combining these variables to evaluate their diagnostic performance. Statistical significance was defined as a *p*-value < 0.05. Additionally, a multivariate-adjusted smoothing spline was generated to assess the relationship between the SI and T-score of BMD, using generalized additive models in R statistical software (version 3.62; R Foundation for Statistical Computing, Vienna, Austria). It is a method used in statistical analysis to model nonlinear relationships in data, adjusting for the effects of multiple variables to smoothly express the relationships between them. This allows us to observe that as the SI value increases, the T-score tends to increase. The analysis was conducted using R Studio version 1.2.5033 (PBC, Boston, MA, USA). For data visualization and graphical representation, the ‘ggplot2’ library was employed within the R environment.

## 3. Results

The non-osteoporotic group had a mean age of 69.58 years, while the osteoporotic group had a mean age of 67.14 years. No statistically significant differences were observed in terms of sex distribution, BMI, or various comorbidities, including hypertension, DM, hyperlipidemia, cerebrovascular accident, liver cirrhosis, angina, asthma, cancer, and hemodialysis between the two groups. However, there were statistically significant differences noted in both the traditional and new SI, with the non-osteoporotic group having higher mean values of 117.57 and 100.54, respectively, compared to the osteoporotic group, which had mean values of 80.83 and 68.32, respectively. Additionally, there was a tendency towards lower index values and a tendency for sarcopenia in the osteoporosis group (Table 1).

The comparison between the two groups divided by the median values of the traditional and new SI revealed the following results. First, in the comparison between the low traditional SI and high traditional SI groups, there was a statistically significant difference in the T-score values from the BMD test, with mean values of −2.64 and −1.95, respectively, indicating that patients in the low traditional SI group had lower T-scores. Additionally, the distribution of DM was statistically different, with 37 patients in the low traditional SI group and 25 in the high traditional SI group, showing a higher prevalence of DM in the low traditional SI group. No other variables showed statistically significant differences (Table 2). Similarly, when comparing the low new SI group to the high new SI, a statistically significant difference was found in T-score values, with mean values of −2.62 and −1.97, respectively, indicating that patients in the low new SI group had lower T-scores. The distribution of DM was also statistically different, with 36 and 26 patients in the low and high new SI groups, respectively, showing a higher prevalence of DM in the low new SI group. No other variables showed statistically significant differences (Table 3).

A multivariable logistic regression analysis was conducted for each SI based on the presence or absence of osteoporosis, and it revealed statistically significant results for three variables: traditional and new SI, sex, and DM. In the analysis using traditional SI, the respective odds ratios were found to be 0.906 for the traditional SI, 9.498 for females, and 1.186 for patients with DM, which indicates that lower traditional SI values are associated with decreased osteoporosis. In contrast, being female and having DM was associated with increased odds of developing osteoporosis. In the analysis incorporating the new SI, the odds ratios were 0.906 for new SI, 1.148 for females, and 5.90 for patients with DM, respectively (Table 4 and Table 5).

**Figure 2:**
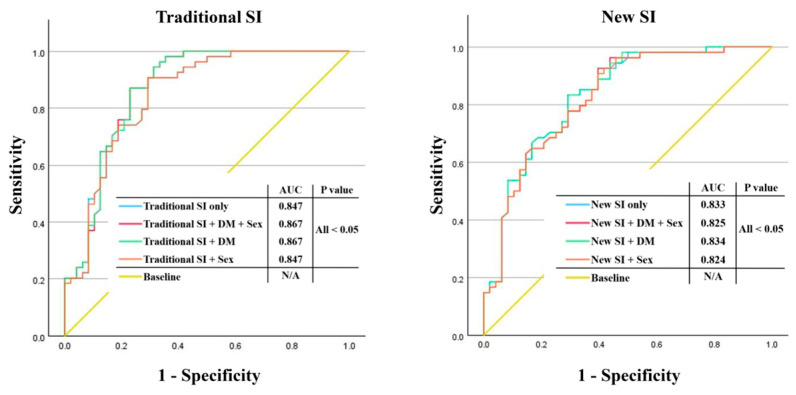
ROC curve and AUC. ROC curves for different variables that were statistically significant in the multivariable logistic regression analysis. All models demonstrated satisfactory performance with an AUC of 0.8 or greater. While there were no notable differences in AUC among all cases, the AUC was typically marginally higher when employing the traditional SI. (1) Index alone (traditional or new SI): blue line. (2) SI and DM: green lines. (3) SI and sex. (4) Red lines indicate SI, sex, and DM. ROC, Receiver operating characteristic; AUC, Area under the ROC curve; SI, Sarcopenia index; DM, Diabetes mellitus.

**Figure 3:**
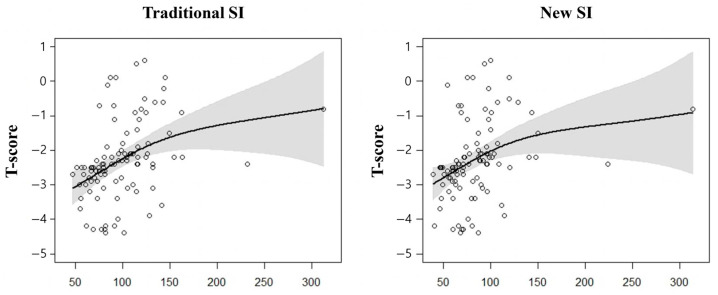
Multivariate adjusted smoothing spline. Multivariate adjusted smoothing spline of two indices (Traditional SI or New SI) according to the BMD T-score showed that as the SI value increases, the T-score tends to increase in both indices. Shaded areas represent 95% confidence intervals. SI, sarcopenia index; CI: Confidence interval; BMD: Bone mineral density.

The ROC curve was examined by incorporating three variables identified as statistically significant in the logistic regression analysis for diagnosing osteoporosis, and all models showed a satisfactory performance with an area under the curve (AUC) of 0.8 or higher. Although there was no significant difference in the AUC across all cases, the AUC was generally slightly higher when using the traditional SI (Figure 2). Finally, a multivariate-adjusted smoothing spline was employed to confirm the relationship between the T-score, SI, and the new SI (Figure 3).

## 4. Discussion

This study was designed to explore the complex relationship between osteoporosis and SI in individuals diagnosed with CKD. The SI is recognized as one of several methodologies for the assessment and detection of sarcopenia. Through a series of analyses, we demonstrated that both traditional SI and new SI were significantly associated with osteoporosis when applied using diverse evaluation methods in patients with CKD. These findings suggest that the SI may serve as a useful marker for identifying and predicting osteoporosis within this vulnerable population, particularly when incorporated into broader diagnostic frameworks.

CKD is a chronic and progressive disorder characterized by a steady decline in renal function over time. Its systemic impacts extend far beyond the kidneys, affecting bone health through intricate and multifactorial mechanisms. Osteoporosis, a condition defined by decreased bone mass and structural deterioration, emerges as a common and serious complication in CKD patients [3]. This is primarily attributed to a combination of hormonal imbalances, mineral metabolism abnormalities, and disturbances in bone remodeling [3,4]. Elevated parathyroid hormone (PTH) levels, a hallmark of secondary hyperparathyroidism in CKD, drive excessive bone resorption, weakening bone integrity. Simultaneously, CKD is often associated with vitamin D deficiency, which further impairs calcium homeostasis and exacerbates bone loss [4]. Metabolic acidosis, another consequence of advanced CKD, introduces additional challenges to bone health [3]. Acidosis results from the accumulation of acidic metabolic byproducts due to the impaired ability of the kidneys to excrete hydrogen ions [3]. This condition stimulates the release of calcium and phosphate from bones to buffer the blood’s pH, ultimately leading to osteopenia and increased fracture risk [3]. Beyond these metabolic disruptions, CKD directly interferes with bone remodeling processes, reducing the ability of bone tissue to undergo normal turnover [3,16]. Among CKD-associated bone disorders, adynamic bone disease is particularly noteworthy [3,16]. Characterized by a profound reduction or absence of bone formation and turnover, this condition exemplifies the broader category of renal osteodystrophy and underscores the significant skeletal burden imposed by CKD. Collectively, these interconnected pathways illustrate the multifaceted nature of CKD-induced osteoporosis, which arises from the interplay of mineral dysregulation, hormonal perturbations, metabolic derangements, and impaired bone dynamics [3,4].

In this context, the potential utility of SI is notable, as its calculation relies on serum creatinine and cystatin C, biomarkers routinely measured in CKD patients to assess renal function. Traditionally, various methods have been used to assess muscle mass, including magnetic resonance imaging, computed tomography, and bioelectrical impedance analysis. However, these methods have drawbacks, such as being time-consuming, uncomfortable, and, in some cases, unreliable. Additionally, some techniques can be costly and involve exposure to radiation. The evaluation of muscle function often involves measurements of handgrip strength and gait speed, but these assessments typically require specialized equipment or trained personnel and may be prone to measurement errors. Therefore, the ability to assess sarcopenia using only the SI calculation in CKD patients is highly convenient and can be applied effectively in clinical practice. Additionally, as shown in this study, the SI value can even predict the degree of osteoporosis, making it useful.

In recent years, growing attention has been directed toward the broader clinical applicability of the SI across a range of medical conditions [7,9,17]. The traditional SI, derived from the ratio of serum creatinine to cystatin C, has been investigated in numerous studies and shown to correlate with a variety of clinical outcomes [12,13]. For example, it has been linked to prognostic implications in cardiovascular diseases, where lower SI values are associated with adverse events [12], as well as in chronic obstructive pulmonary disease (COPD), where SI has been used as a marker for respiratory failure [13]. Studies focusing on middle-aged and elderly populations have revealed significant associations between SI and mortality risk, with lower SI values predicting higher mortality rates [18]. Conversely, in hospitalized elderly patients, higher SI levels have been associated with reduced risks of malnutrition and sarcopenia, suggesting a protective role in this context [19]. Moreover, the SI has demonstrated utility in predicting negative outcomes in highly specific clinical scenarios. For instance, in patients undergoing transcatheter aortic valve replacement, the SI has been used as a marker for predicting adverse outcomes [20]. Similarly, in older adults with stage III or IV non-small cell lung cancer, the SI has been linked to survival outcomes [21].

The timed urine creatinine value depends solely on muscle mass when renal function is stable. The eGFR based on serum cystatin level is a better method for estimating renal function in patients with low muscle mass than creatinine [15]. For this reason, the new SI proposed by Lien has emerged as a promising alternative. This new SI has shown stronger correlations with measures of muscle strength and skeletal muscle mass, particularly in patients with advanced cancers, than the traditional SI [22]. Currently, there is a lack of sufficient research on the new SI, and no studies have been conducted to determine which SI is a better indicator. Therefore, it is hard to say which SI should be prioritized.

Regarding the diagnosis of sarcopenia, there is no single universally accepted threshold for diagnosing sarcopenia using the SI. Likewise, although CT-derived muscle area measurement is used previously in sarcopenia research, there is not a universally accepted cutoff value for diagnosing sarcopenia. However, some studies have suggested cutoff values. The values suggested by Martin et al. [23] divided patients according to BMI and sex for skeletal muscle index (SMI). In patients with a BMI < 25 kg/m^2^, sarcopenia was defined as SMI < 43 cm^2^/m^2^ for males and <41 cm^2^/m^2^ for females. Similarly, for patients with a BMI > 25 kg/m^2^, the cutoff values for sarcopenia were <53 cm^2^/m^2^ for males and <41 cm^2^/m^2^ for females. Prado et al. suggested cutoff values of 52.4 cm^2^/m^2^ for males and 38.5 cm^2^/m^2^ for females [24]. In the study by Barreto et al. [7], abdominal CT scans were used to measure the para-spinal muscles at the L3/4 vertebral level, and this showed a significant correlation with the traditional SI. In this study, the average skeletal muscle surface area was 124 ± 33 cm^2^, the average SMI was 42.4 ± 9.8 cm^2^/m^2^, and the average SI was 77 ± 25 [7]. If an SMI value of approximately 40 is used as a cutoff for defining sarcopenia, considering the results of the above study, it seems that a traditional SI around 65–70 could serve as the cutoff for diagnosing sarcopenia. Based on our study and considering the difference between the new SI and traditional SI, the cutoff value for the new SI would likely be around 50–55. However, these values are not scientifically derived through statistical analysis, so further analysis and research will be necessary in the future to validate them. Additionally, based on the results of this study, it is believed that the degree of osteoporosis could be inferred according to the values of the traditional SI and new SI.

Several studies have shown an association between BMI and osteoporosis. Generally, as BMI increases, the risk of osteoporosis tends to decrease [25,26]. However, in our study, the BMI was similar between the two groups (osteoporotic and non-osteoporotic) despite the osteoporotic group having lower muscle mass (as indicated by the low SI), which can be explained by the fact that BMI is a measure based on both weight and height, but it does not differentiate between the components of body weight (e.g., fat, muscle, and bone mass). Therefore, individuals in both groups might have similar overall body weight, but the distribution of fat, muscle, and bone mass could be quite different.

In our study, further analysis revealed significant risk factors for osteoporosis, with female sex and DM emerging as particularly noteworthy predictors in the multivariable logistic regression model. The association between female sex and osteoporosis is well established in the literature, reflecting the disproportionately higher prevalence of the condition among women compared to men [27]. This disparity is largely driven by estrogen deficiency, which accelerates bone resorption and decreases BMD following menopause [27]. Estrogen plays a critical role in maintaining skeletal homeostasis by regulating osteoblast and osteoclast activity, and its absence disrupts this balance and predisposes women to osteoporosis [27]. Similarly, DM was identified as a significant risk factor for osteoporosis in our analysis. The relationship between DM and bone health is complex and multifaceted. In type 1 DM, patients generally exhibit lower BMD due to factors such as insulin deficiency and reduced bone formation [28,29]. While patients with type 2 DM may initially present with normal or even elevated BMD, this apparent protective effect diminishes over time as bone quality deteriorates, increasing fragility and fracture risk [29]. Mechanistically, DM impairs osteoblast function, enhances osteoclast activity, and promotes the accumulation of advanced glycation end products (AGEs) in bone collagen, which compromises bone strength [28]. In the comparative analysis between the two groups, the lower SI group demonstrated a significantly lower BMD, and the prevalence of DM was considerably higher in this group, further supporting the association between osteoporosis and DM.

This study, although valuable, has some limitations. First, it only included Asian subjects. Ethnic differences can influence health outcomes; therefore, caution should be exercised when extrapolating these findings to other populations. Second, this study utilized cross-sectional data, which provided a snapshot at a single point in time. Consequently, causation cannot be established, and long-term correlations or outcomes cannot be definitively confirmed. Given these limitations, it is important to interpret the results of this study with caution and consider the need for further studies with larger and more diverse populations to validate and expand upon these findings. Third, this study was conducted in a cohort of patients with CKD. Future studies should conduct similar investigations in other patient populations to further elucidate the relationship between SI and osteoporosis. Such studies would enhance our understanding of these conditions across various clinical contexts and may inform more comprehensive management strategies. This study underscores the importance of SI as a potential tool for evaluating sarcopenia and osteoporosis in CKD patients. However, further research involving larger, more diverse populations and longitudinal data is essential to validate these findings and extend their applicability. Such efforts could pave the way for more effective and personalized approaches to managing bone health in CKD and beyond.

## 5. Conclusions

This study confirmed that both the traditional and new SI are associated with osteoporosis in patients with CKD.

- Traditional SI = Serum Creatinine [mg/dL]/Serum Cystatin C [mg/L] × 100
- New SI = Serum Creatinine [mg/dL] × Cystatin C-based estimated GFR (eGFR_CysC) [mL/min/1.73 m^2^]

Consequently, clinicians may utilize a simple calculation based on the SI to estimate bone health in patients with CKD, making it a valuable tool in clinical practice, even in the absence of advanced radiological examinations.

## Figures and Tables

**Table 1:** Patient demographics between the non-osteoporotic and osteoporotic group.

	Unit	Non-Osteoporosis (N = 54)	Osteoporosis (N = 48)	*p* Value
**BMD (T-score)**		−1.54 ± 0.89	−3.15 ± 0.64	**0.001**
**Age**	year	69.58 ± 10.6	67.14 ± 12.69	0.063
**Sex ^a^**	Male/Female	14/40	10/38	0.447
**BMI**	kg/m^2^	24.18 ± 4.69	23.24 ± 5.19	0.444
**Traditional SI**		117.57 ± 38.92	80.83 ± 22.38	**0.009**
**New SI**		100.54 ± 41.29	68.32 ± 18.77	**0.008**
**Comorbidities ^b^**				
Hypertension		37	31	0.893
DM		35	27	0.551
Hyperlipidemia		5	3	0.556
CVA		16	15	0.909
Hemodialysis		6	4	0.594
Liver cirrhosis		3	0	0.096
Angina		21	14	0.596
Asthma		6	2	0.186
Cancer		8	4	0.297
**Laboratory test**				
Cystatin C	mg/L	2.49 ± 1.86	2.39 ± 1.63	0.793
GFR_Cystatin C		50.07 ± 30.27	46.58 ± 22.17	0.304
Creatinine	mg/dL	3.16 ± 3.39	1.94 ± 1.46	**0.022**
GFR_Creatinine		34.26 ± 22.81	45.39 ± 25.98	0.527

BMI, Body mass index; SI, Sarcopenia index; DM, Diabetes mellitus; BMD, Bone mineral density; CVA, Cerebrovascular accident; GFR, Glomerular filtration rate. **^a^** Pearson’s chi-square test, **^b^** Mann–Whitney U test, otherwise by *T*-test. Data were expressed by mean ± standard deviation. **Bold** in the *p* Value means the *p* Value < 0.05.

**Table 2:** Patient demographics based on the traditional SI (low and high group).

	Unit	Low (N = 51)	High (N = 51)	*p* Value
**BMD (T-score)**		−2.64 ± 1.05	−1.95 ± 1.09	**0.001**
**Age**	year	65.59 ± 11.23	69.24 ± 10.05	0.078
**Sex ^a^**	Male/Female	13/38	11/40	0.641
**BMI**	kg/m^2^	23.91 ± 5.01	23.60 ± 4.76	0.390
**Comorbidities ^b^**				
Hypertension		29	39	0.077
DM		37	25	**0.010**
Hyperlipidemia		3	5	0.479
CVA		16	15	0.778
Hemodialysis		5	5	0.998
Liver cirrhosis		1	2	0.570
Angina		20	15	0.264
Asthma		2	6	0.149
Cancer		6	6	0.971

BMI, Body mass index; SI, Sarcopenia index; DM, Diabetes mellitus; BMD, Bone mineral density; CVA, Cerebrovascular accident; GFR, Glomerular filtration rate. **^a^** Pearson’s chi-square test,**^b^** Mann–Whitney U test, otherwise by *T*-test. Data were expressed by mean ± standard deviation. **Bold** in the *p* Value means the *p* Value < 0.05.

**Table 3:** Patient demographics based on the new SI (low and high group).

	Unit	Low (N = 51)	High (N = 51)	*p* Value
**BMD (T-score)**		−2.62 ± 1.00	−1.97 ± 1.00	**0.002**
**Age**	year	66.37 ± 10.05	68.45 ± 10.60	0.055
**Sex ^a^**	Male/Female	14/37	10/41	0.350
**BMI**	Kg/m^2^	23.26 ± 4.72	24.11 ± 5.11	0.219
**Comorbidities ^b^**				
Hypertension		31	37	0.258
DM		36	26	**0.030**
Hyperlipidemia		4	4	0.977
CVA		15	16	0.881
Hemodialysis		5	5	0.999
Liver cirrhosis		1	2	0.570
Angina		19	16	0.484
Asthma		2	6	0.149
Cancer		6	6	0.971

BMI, Body mass index; SI, Sarcopenia index; DM, Diabetes mellitus; BMD, Bone mineral density; CVA, Cerebrovascular accident; GFR, Glomerular filtration rate. **^a^** Pearson’s chi-square test, **^b^** Mann–Whitney U test, otherwise by *T*-test. Data were expressed by mean ± standard deviation. **Bold** in the *p* Value means the *p* Value < 0.05.

**Table 4:** Odds ratios for developing osteoporosis based on the traditional SI.

Variables	Odds Ratio (95% CI)	*p* Value
Traditional SI	0.906 (0.821–0.984)	0.001
Sex *	9.498 (5.942–15.132)	0.03
DM	1.186 (0.687–1.801)	0.048

The other variables were not statistically significant. SI, Sarcopenia index; CI, Confidence interval. * Compared to a male patient.

**Table 5:** Odds ratios for developing osteoporosis based on the new SI.

Variables	Odds Ratio (95% CI)	*p* Value
New SI	0.906 (0.803–0.997)	0.001
Sex *	1.148 (0.793–1.621)	0.031
DM	5.9 (2.931–8.213)	0.049

The other variables were not statistically significant. SI, Sarcopenia index; CI, Confidence interval. * Compared to a male patient.

## Data Availability

The data that support the findings of this study are not openly available for reasons of sensitivity but are available from the corresponding author upon reasonable request.
